# Next Generation Networks: Featuring the Potential Role of Emerging Applications in Translational Oncology

**DOI:** 10.3390/jcm8050664

**Published:** 2019-05-11

**Authors:** Enrico Capobianco

**Affiliations:** Center for Computational Science, University of Miami, Miami, FL 33146, USA; ecapobianco@med.miami.edu; Tel.: +1-3052434692; Fax: +1-3052439304

**Keywords:** networks, modularity, entropy, controllability, synchronization, symmetry, cancer metastasis

## Abstract

Nowadays, networks are pervasively used as examples of models suitable to mathematically represent and visualize the complexity of systems associated with many diseases, including cancer. In the cancer context, the concept of network entropy has guided many studies focused on comparing equilibrium to disequilibrium (i.e., perturbed) conditions. Since these conditions reflect both structural and dynamic properties of network interaction maps, the derived topological characterizations offer precious support to conduct cancer inference. Recent innovative directions have emerged in network medicine addressing especially experimental omics approaches integrated with a variety of other data, from molecular to clinical and also electronic records, bioimaging etc. This work considers a few theoretically relevant concepts likely to impact the future of applications in personalized/precision/translational oncology. The focus goes to specific properties of networks that are still not commonly utilized or studied in the oncological domain, and they are: controllability, synchronization and symmetry. The examples here provided take inspiration from the consideration of metastatic processes, especially their progression through stages and their hallmark characteristics. Casting these processes into computational frameworks and identifying network states with specific modular configurations may be extremely useful to interpret or even understand dysregulation patterns underlying cancer, and associated events (onset, progression) and disease phenotypes.

## 1. Introduction

### 1.1. Background

Cancer research is in part concerned with establishing the governing rules of systems subject to complex dynamics. Such systems have become increasingly data-intensive and call for machine learning (ML) approaches for modeling such dynamics. Despite such need and for a variety of reasons the power of statistical learning is rarely exploited in full in biomedical applications. The two main effects are (a) learning algorithms may offer no guarantees of generalization, and (b) discoveries derived from the assembly of a variety of experimental and computational resources and tools may need more complex validation. In general, our ability to process new information through specialized data-driven analytic tools efficiently separating noise and algorithmic artifacts from real data features may be diminished by the limited capacity of developing transferable knowledge due to a lack of generalization. 

Multiple factors must be considered to explain carcinogenesis as a paradigm of complexity. Both endogenous factors, say DNA replication or cellular interactions, and exogenous ones, say lifestyle, diet and environmental influences, are increasingly studied together in view of the pervasiveness of big data and electronic health records, the adoption of sensor and wearable technologies, and the methodological advances made possible through Artificial Intelligence (AI), machine and deep learning and networks. Both the abundance and the variety of factors influencing cancer heterogeneity characterize the inherent complexity of this disease. For instance, such factors may affect therapy response [1]. Identifying response biomarkers is critical for the assessment of therapy effects and patient monitoring, being a central component of patient outcome trajectories (see [2,3], among others). 

Redundancy in cancer may be seen as an obstacle to therapies [1]. Intuitively, when various biological system’s components act similarly, inhibiting one or a few of them has only limited impact on therapy. Although redundancy may implicate the presence of cooperative dynamics between multiple bioentities, especially genomics, studies emphasize through gene profiles and pathway landscapes a few inherent limitations [4]. Both compositional (large intersections between signatures) and functional (different transcriptional read-outs relative to the same biological process) redundancy appear from cancer gene signatures, while usually only a fraction of all possible genomic measurements can be then validated. With reference to cancer genomes, complexity shows through differentiated somatic alterations of normally functional cells between and within cancer types [5]. Functional redundancy is fundamental to establish biological robustness, a property that allows internally and/or externally perturbed systems to maintain their functions [6]. The relationship between redundancy and robustness has also been described quantitatively [7]. From a translational standpoint, relatively few examples are usefully available to justify either biological or clinical validation purposes. In fact, most experimental validation techniques, including clinical trials, are not designed for high-throughput redundant analysis. Overall, this constraint binds the possibility of determining suitable candidate genes or proteins playing the role of putative diagnostic biomarkers or therapeutic targets. 

In view of the importance assigned to the search of new types of integrative genomic signatures, beyond the various gene lists proposed in cancer studies, networks provide a variety of inference strategies aimed at uncovering complex regulations and suggesting possible interventions (see [8,9,10], among many other seminal papers). In such regards, understanding and controlling network dynamics are essential tasks that remain largely unexplored in oncology. In particular, investigating network dynamics might be highly relevant for the study of change of a system’s status through time, a condition which can be naturally referred to metastatic processes. Additionally, cancer dynamics could be suitably monitored by networks with reference to both structural aspects (topological node characteristics) and functional aspects (collective node behavior captured by modules in response to perturbations) (see [11,12,13,14,15], for instance).

### 1.2. Networks

‘Next generation networks’ is a term here used to address selected innovative directions in network medicine with a potential impact on personalized/precision/translational oncology. Identifying network states and specific modular configurations that may reveal dysregulations typical of cancer-associated events (onset, progression) and phenotypes is an emerging driver of translational oncology applications [10]. Here too the goal of the proposed use cases and their related analyses is to leverage network properties that appear still largely unutilized in oncology but are relevant for metastatic processes, for instance to understand how such dynamics progress stage-wise while being driven by well-known cancer hallmarks. 

A complex property typically relevant for cancer treatment is synergy. Synergistic interactions occur in a variety of cancer contexts, and involve genes, proteins, drugs etc. [16]. First, synergies are usually quite hard to detect [17]. Second, they do not allow straightforward analytical treatment (especially three-way and above). In principle, exploiting synergy in a complex system means that the overall redundancy effects are somewhat neutralized. This is usually due for two reasons: (a) synergy inherently casts system’s information into a reduced number of components. In other words, dimensionality reduction performs feature selection as an essential step towards an increased efficiency; (b) redundancy yields spurious system’s correlations among an individual system’s components, and this translates into insufficient accuracy to ensure robustness and consistency. A major role for synergy is expected in biological systems whose gene regulatory dynamics, metabolic pathways, protein markers are relevant to clinics. In each of such contexts the use of pairwise associations cannot ensure a global understanding of complex dynamics. Nevertheless, despite the need of identifying synergistic regulation or dysregulation for inference scopes, the identifications often remain computationally intractable. Therefore, the analysis of pairwise relationships may at that point represent a feasible although only approximate solution to an optimal one.

Networks also offer the opportunity to study co-existing redundancy and synergy. This can be seen specifically in the cancer context with reference to redundant signature gene sets that nevertheless significantly enrich pathways. When considering networks as modular architectures, it is expected that the identified modules (or communities, or sub-networks) are significant because functionally associated. In cancer, modules can point to processes (such as metastasis) in which states reflecting the cell conditions can be defined. With big data, the recourse to discrete state approaches may still be computationally expensive, but the advantage is that various cell types (cancerous and non) can be monitored through state transitions, depending on the change of conditions (due to stressors, therapies etc.). 

Intuitively, cell state dynamics could be studied by discrete-time Markov models as elementary stochastic processes modeling the cell state evolution over time, and specifically by establishing dependence of an outcome at time *n* on the previous outcomes although with limited memory *k* steps back in time. Knowledge of the transition rates for healthy and diseased cells could be important for (i) understanding disease mechanisms, (ii) estimating the temporal effect of a certain perturbation or stressor, and (iii) predicting cell state trajectories at given times. More sophisticated methods lead to the so-called combinatorially dysregulated subnetworks, in which system decompositions are used to identify the most phenotypically informative states [18,19]. The most common way to quantify the effects of synergistic dysregulation uses differential expression. This choice may appear reductive in view of other possible biological entities representing complex structures and requiring other types of measurement. In practice, while simple measures are routinely used to assess functional similarity in biological clusters and pathways, networks display highly interconnected modular configurations that legitimate the importance assigned to coordinated actions underlying disease phenotypes, including cancer ones. 

### 1.3. Cancer Networks Taxonomy

Network tools have been widely used to infer cancer system dynamics due to their ability to represent the complexity of structural and functional cancer characteristics. This aspect combines well with the ability of visualizing integrated experimental, computational and clinical outcomes. To this end, networks effectively synthesize different types of data (qualitative and quantitative) as well as inputs from various data generating processes, such as the ones linked to genomic, metabolomic, proteomic and imaging sources. Therefore, networks may play a role in guiding the discovery process in cancer research and ultimately deliver computationally efficient, verifiable and highly interpretable solutions that naturally combine the results of experimental and modeling cycles.

In parallel with what can be observed in many other application fields, a taxonomy of networks applicable to cancer is gradually taking place following the advances in domains such as epigenetics, non-coding RNA, imaging, just to mention a few. These ‘next generation cancer networks’ involve novel developments of algorithms and inference methods in response to emerging interdisciplinary applications. Namely, a series of topics potentially relevant to translational oncology are: (a) optimizing network design and functionality, (b) measuring the influences of physical network structural characteristics in order to achieve robust configurations, (c) cross-layering algorithms for in-depth (multiresolution) understanding of cellular networks, (d) leveraging the interactions between multitype networks while balancing their stability, (e) processing information packets instead of single entities, (f) surveying anomaly detection approaches and monitoring early warnings, (g) Facing the increasing heterogeneity of multi-sourced data contexts. All such topics benefit from several ongoing theoretical and methodological advances that in principle may improve network inference but also require systematic testing to establish significance in diagnostics, targeted therapy, drug repurposing, toxicity, etc. (see [20,21,22], for instance). Cancer contexts already mature for such applications include prevention, in which the enhancement of risk assessment strategies based on data multitudes enables clinicians to identify at-risk populations prior to disease onset. Then, another high-impact area is epigenetics covering the spectrum of perturbation mechanisms referred to biological and functional effects of small epigenetic changes (e.g., DNA methylation, histone modifications, and non-coding RNA expression) and the epigenetic events longitudinally impacting health outcomes. 

Aiming at further generalizing cancer network applications, the emphasis in this work is assigned to three network properties: *entropy, symmetry, controllability.* They share the characteristic of having a substantial amount of unexplored potential. Entropy, surely the most studied property so far, is associated with the quantification of the uncertainty influencing the discovery process. Symmetry can be associated with system robustness and resilience. Controllability, when translated in biomedical networks, may translate into the ability of performing feasible and accurate gene/protein selection and improving marker/target prioritization. As anticipated before, the multifaceted cancer metastatic process is here chosen as the ideal ground for applications presented through use cases and centered on the three mentioned network properties. 

## 2. Methodological Themes

### 2.1. On Entropy

There are many possible ways to utilize entropy in complex biological systems, and cancer entropy has been a topic widely covered [23,24]. At a formal level, an interesting starting point for describing the complexity of a system is offered by the concept of *set complexity* [25,26,27]. This is coherent with the idea that a complexity measure for a multivariable system is derived from a quantity initially computed over subsets of the variables. Set complexity for two network nodes (i,j) is defined as follows:

(1) Ψ=c∑i∑jmax{S(i),S(j)} MI(ij)[1−MI(ij)]

where S represents the *Shannon entropy*, or S = −log(N)^−1^∑i p_i_ log(p_i_), and MI is the normalized mutual information, or MI = log(N)^−1^∑_ij_ p_ij_ log p_ij_ (p_ij_/p_i_ p_j_). In general, a set of variables may be conceived to be subject (or not) to dependence in collective terms, i.e., all set members contribute to some extent to the prediction of one of them. This sort of pattern is useful for interpreting network constraints of functional relevance from a biological viewpoint. In genomics, this might be relevant for capturing nonlinear or high-order interactions underlying highly complex regulation signatures that simple gene lists cannot represent. This concept also implies that biological networks presenting modular architectures can maximize the set complexity. Modularity basically enforces the connectivity between nodes and represents a structural feature, one quantitatively reflected in the MI measure whose advantage is to detect significant functional dependence beyond simple correlation. 

In the presence of nonlinear dynamics, things become more complicated and basins of attraction are used to link a set of condition from initial to final states. The concept of *basin entropy (bS)* allows to quantify the uncertainty inherent to such state transitions by probing network behavior with varying parameters [28]. This helps quantifying the possible unpredictability of the dynamical system underlying a network [29]. For gene regulatory networks, *bS* can be defined by considering the basins as the state space components, as follows [30]:

bS = −∑_I_ s_i_ log s_i_ (2)

where the subscript *s_i_* indicates the size of the basin *i*. Notably, bS is minimal when one basin has all the states and maximal when the states are distributed into different attractors.

Network states may well represent dynamic entities fluctuating between states and the possibility of monitoring all such transitions between states allows to define trajectories. Unlike in deterministic systems, in stochastic systems these trajectories can exhibit multiple dynamical regimes. As states may also recur, attractor states can be formed (namely, an attractor is where a system tends to go by evolving from various starting conditions). A basin of attraction is generated when multiple states tend to flow into attractors. It is important to note that the size of an attractor is contributed by the number of basin states gravitating around it. Entropy can be computed for these types of states, and because state-space partitions are non-unique for a given network, the related state entropies are also non-unique. This offers a rationale for relying on average entropy measures, i.e., computed on network ensembles. Average entropies refer to the *fluctuation theorems* [31]. These describe systems’ non-equilibrium states analytically, through the statistical fluctuations in time-averaged properties. Equivalently, the system’s resilience (robustness) R correlates with uncertainty, hence entropy, according to:

dR dS > 0(3)

### 2.2. Metastasis and Its Stochastic Dynamics

In general, an entropic increase may depend on the robustness of cancer cells to perturbing factors or environmental stressors, including the tumor microenvironment [32]. Interestingly, differential expression tends to anticorrelate with differential network entropy [33]. For example, overexpressed proliferation genes (carrying oncogenes) were found to be associated with reduced network entropy across several cancers. This is very relevant to infer the metastatic potential at early stages of disease progression. It was then established that the analysis of transcriptional signatures associated with perturbed cancer-related genes reveal metastatic cancer subtype states such as epithelial to mesenchymal transition or EMT-like associated to an inflammation state, and proliferative state (increased metabolism and systemic stress signaling) [34]. Typically, EMT transitions occur in cell morphology with a corresponding reduction of cell function synchronization and increased motility levels. Another possible finer subtype derived from the adaptive stress signaling could be referred to the so-called phenotype switching, which occurs after the inhibition of receptor (non-receptor) kinases, or in response to (chemo-) therapy. Most importantly, signaling events occur not only dynamically but rapidly in cancer cell that respond to therapy, and then can turn to survival or not depending on resistance. Adaptive stress signaling has been indicated as the reference emerging research area to such developments [35]. 

Metastasis evolves over time and in response to therapy. Therefore, cancer might in principle be modelled according to a stochastic sampling process, and the genotypes would be the result of random drawing from mutational pooling, this latter acting as the process underlying the data generating mechanism [36]. A mutation ensemble would thus be composed of various states to which perturbed networks can be associated depending on the probability of state activation or repression following a certain mutation. Under conditions of equilibrium, or stationarity, the observed experimental data endowed with the phenotypic response (metastatic or non) can be considered the outcome of ensemble averages operating over many states. These states have differentiated entropy degrees, and because they interact between them and with the environment, the influences at systems level fluctuate depending on entangled versus decoherent conditions. Only in the former scenario the total state cannot be factorized as a product of component states, which basically differentiates the role of interaction in the two conditions. 

A stochastic matrix can be formed by the transition probabilities between states X_i_ and X_j_, assuming they are constant over time and depend only on the reference state. This way, the transition matrix would define a discrete-time Markov process explaining state evolution dynamics of the population of cells under consideration [37,38]. At a network scale, a dynamic rewiring occurs through the node interactions determining the network state trajectory. Under no change of state over time, an attractor may be observed (or an attractor landscape if all possible state trajectories are considered). Both genetic and epigenetic alterations can underlie such a network evolution process, and the genomic instability and/or loss of proteostasis may explain uncontrolled cell proliferation [39].

A steady-state uncertainty measure of the information flow is provided by the network entropy computed from the entropy rate ∆S [40]:

∆S = ∑_i_π_i_ S_i_ (4)

considering the stationary distribution of the transition probabilities π and with the entropies S_i_ computed locally (at each state). A change in entropy rate would imply that the effects of signaling are likely present in response to some type of perturbation. 

### 2.3. Therapy and Phase Transitions

While an interesting interpretation has been offered in a context of primary and metastatic versus non-metastatic cancer, showing that in the former case the entropy increases and thus becoming an indicator of cancer progression, a finer formulation would be required to consider network nodes instead of the states. The local entropy for a given node would be a special case:

S_i_ = −[log(k)]^−1^ ∑_i_ p_i_ log p_i_ (5)

with *k* as the degree of the reference node i, and *p_i_* as the probability linking the reference node with other nodes. An important consequence is that cancer phenotypes can be assessed by their local entropies from degree computed node-wise. The related distribution allows to assess network heterogeneity, and how randomness characterizes metastatic versus non-metastatic networks. 

A principled elucidation of the dynamical responses of cancer networks naturally involves therapy, and the starting point is measuring the differential gene expression that determines phenotype-driven network configurations. Under the influence of external factors, a phase transition may occur once certain critical levels are reached. A normal cell can undergo a transition and thus become not apoptotic due to cumulated mutations too. Epigenetics play a central role in such regards [41]. However, relevance goes to genome stability too, in parallel and in light of genomic rearrangement studies elucidating concepts like chromothripsis and chromoplexy that underlie catastrophic events. In general, some types of compensatory perturbations might be designed and used to direct a network to a desirable state, thus deviating from undesirable ones (this topic is re-considered below, when control arguments are discussed). 

While a phase space represents all possible states in a dynamical system, it is important to note that biological systems tend to be sparse, with a high number of degrees of freedom reflected over the big set of parameters characterizing the system [42]. Similarly, complexity is generated by the interactions among bioentities like proteins (many are false negatives or missing values can be present) or gene profiles (only in part significant due to noisy signals and presenting multiple transcripts). Depending on the possibility of limiting the overall uncertainty, this complexity can be mitigated by dimensionality reduction aimed at preserving the structural information. Due to sparsity, the local structures are those that usually orient the phase space while the system evolves. For example, multiple evolutionary trajectories are observed in cancers even before achieving a transformed state, such as a metastatic one, thus establishing the importance of the temporal dimension of cancer heterogeneity. 

### 2.4. On Symmetry

The role of symmetry in cancer biology is multifaceted, therefore complex. Biological signals may cause a shift in the normal balance of symmetric and asymmetric cell division, which triggers the differentiation arrest observed in cancer progression [43]. Studies on symmetric stem cell divisions have considered homeostasis [44]. While asymmetric division is very important in generating diversity during development, its dysregulation is relevant for promoting oncogenesis. It is well-known that a small fraction of cancer stem cells or cancer-initiating cells in the affected cell populations is resistant to many anticancer drugs and produces many heterogeneous tumor cell populations. As a result, tumor recurrence and metastasis become more likely. Cancer stem cells undergo asymmetric cell division, something physiological in normal stem cells [45,46]. While occurring during development and tissue homeostasis, this enables a balance between self-renewal stem cells and differentiated cells in a single division, an in turn between the stem cell pool and the progenitor cell pool [47,48]. 

Coming to the problem of how to represent symmetry, networks offer a natural mathematical framework for studying symmetry. For instance, they might reveal how symmetry signatures shape network connectivity, thus exerting structural and functional influences. In such regards, a few open questions are:

(1) Do symmetric patterns induce localized network dynamics, i.e., those represented by modules, clusters, motifs? If this is the case, how specific these structures are?
(2) Are the possibly observed patterns robust or fragile with regard to symmetry breaking? The latter would imply the presence of fluctuations leading the system to a critical point, and thus a likely change of state that in cancer might be a signature of metastasis.

Regarding cancer progression, and with reference to possible therapeutic interventions, the latter might enable the addressed fluctuations, although other open questions are:

(1) How the influences exerted by symmetry patterns depend on cancer heterogeneity?
(2) Are such patterns controllable?

At a general methodological level, detecting symmetries may facilitate inference, particularly with multimodal data distributions or in the presence of spurious correlations. There are several types of symmetries, and common symmetries are permutation, scaling and translation ones. Likelihood functions can embed symmetries. Consider the sample data x ϵ X and a model M(x,θ) with parameters θ ϵ θ*. These parameters may be non-identifiable due to the underlying presence of symmetry. Namely, a symmetry ρ: θ → θ is a measurable function that makes θ no longer identifiable from the data x. In such cases, one needs symmetry breaking to augment inference performance. The natural way to obviate to symmetry effects is to change the model parameterization or equivalently to enable a transform θ → ψ, with ψ ϵ ψ*. 

Considering networks as the reference parametric models, symmetries may reveal localized dynamical modes in the data, and more importantly these might be stable or unstable. With respect to stability, symmetry may lead to node reduction without influencing the overall dynamics [49,50]. This is useful for studying network redundancy versus network quotients or skeletons [50,51]. Here, no structural repetition is present but other structural network properties remain, together with the inherent complexity. In general, defining symmetries is something inherent to node permutations. Two nodes are permuted when their links are rewired and a switch occurs. Collecting all nodes into the adjacency matrix A, in which A_ij_ = 1 (if node i and node j are adjacent) or 0 (otherwise), a symmetry is present when a permutation P is applied to A, leaving it unchanged, i.e., PA = A. This establishes a so-called automorphism, indicating that nodes are topologically equivalent if their permutation does not affect the network structure. 

Since nodes may form a symmetric structure, an orbit is defined by a set of structurally equivalent nodes, i.e., all vertices in the same orbit have the exact same degree. In a cancer context this concept could be associated to nodes reflecting similar cancer properties (proliferation, tumorigenicity, invasiveness etc.). Formally, given a set of nodes in a network N and a set of automorphisms A(N) (forming a group), the orbit of a node n ϵ n(N) is the set (see [52]):

O(n) = {λn ϵ n(N) : λ ϵ A(N)}(6)

From a different standpoint, an orbit is the set whose nodes can be obtained from one another by simple permutations in A(N). A symmetric network partitioning of nodes into orbits establishes disjoint equivalence classes for each node, i.e., an *automorphism partition* (AP) [53]. Of interest is the induced entropy, a measure of the network structural heterogeneity, such that for *n* = 1, N:

S^AP^ = −∑_n_ p_n_ log p_n_ (7)

p_n_ = |n_i_|/n^N^ (8)

the latter being associated probabilities (ratios between AP cell nodes and network nodes). The normalized entropy is then defined simply as:

^n^S^AP^ = S^AP^/log N (9)

With reference to spectral theory, the eigenvalues of symmetric structures are decomposable into redundant and non-redundant eigenvalues depending on eigenvectors localized on the symmetric structure or corresponding to nodes of an orbit. Consequently, networks built over orbits rather than simple nodes may directly reduce the overall redundancy. Looking at how network dynamics are regulated by the eigenvalues, regardless the network structure, the system must be studied at steady state or at possible departures from it. A probability measure inserted in the network allows a probability distribution Pd to be defined on its nodes, and a network entropy be used as a measure of the randomness degree in the network:

S^N^(Pd) = min ∑_i_ p_i_ log p_i_ (10)

Entropy implies the importance of the stochasticity of symmetries (see fuzzy symmetries [54,55,56]) based on network ensembles. Furthermore, network communities, which are cohesively connected sets of likely functionally similar nodes, exert also an impact on another important aspect, synchronization [57]. It has been recently shown that cell cycle synchronized transcriptional patterns are highly complex in their functions (DNA replication, cell division etc.) and changes associated with cell cycle transition points that affect regulation of developmental patterns in cancer cells [58]. 

In network terms, synchronization refers to functional interaction between interacting systems states as measured by node dynamics appearing correlated in time due to complex interaction laws and depends on the spectral properties. Network topology can be summarized by the Laplacian, a symmetric matrix in which the second eigenvalue of this matrix may tends to 0, and thus implying that a state of synchronization cannot be reached by the network. An influence comes also from the highest of the eigenvalues. Besides determining, or not determining, the property, no information is available from these two eigenvalues on the relationships with network topology, which is of interest here. Synchronization is enhanced when a high clustering coefficient is measured. This measures the number of neighbors of adjacent nodes, i.e., the closer are the nodes the higher is clustering value. Correspondingly, synchronization is enhanced by the level of node degree heterogeneity and is affected by the ratio between the highest and lowest degree nodes. 

Note that the role of symmetries with reference to network synchronization is investigated in [59,60], while the computational methods to break such symmetries (isolated desynchronization) are discussed in [61]. There are studies in biology justifying how functional similarity refers to symmetry. A typical example is provided by brain areas, for instance when considering neurons from hippocampus and cortex that fire in an oscillatory way and as a network [62]. In general, the construction of functional networks depends on the relationships between their coupling components, which makes synchronization motifs central features. Functional networks are usually heterogeneous, thus representing non-symmetrical structures. This aspect reflects the fact that disruption of the couplings generates symmetry-breaking in the network and also loosens the inherent synchronization motifs distribution. However, if the couplings can sustain synchronization, then symmetry will be characterizing the functional network [63].

### 2.5. On Controllability

Networks evolve through a series of transitions between states, and these may enable changes of structure. State embed conditions in which nodes are, and networks representing complex systems allow to monitor state evolution through the dynamics that regulate the communication between nodes. While a hierarchical structure presents clear directionality, a symmetric structure presents more fluctuations that induce transitions between ordered and disordered conditions. In the analysis of cancer systems, different sets of parameters useful to exert control of transitions between states may be present. They may characterize the cancer type and/or drive the transition from normal to cancer state. Human cancer signaling networks allow node identification (driver or not) and classification according to their role for structural controllability of the network. A common hypothesis is that the nodes critical for achieving centralized control are also therapeutically important. In particular, anti-cancer drugs act primarily against specific genes, i.e., their targets, and networks clearly allow large-scale analysis of therapeutic implications.

Cancer forms a robust system that maintains stable functioning (cell sustenance and proliferation) despite perturbations. Cancer progression occurs stage-wise over time with increasing aggressiveness (and worsening prognosis). The characterization of these stages and identification of the genes driving the transitions are critical steps for the development of effective anti-cancer therapies. In general, cancer robustness can be derived from exchange mechanism between genes at different stages and from different biological processes and/or cellular components. These are all involved in progression stages and allow cancerous cells to evade targeted therapy, therefore suggesting that a possible effective therapy should target a “cover set” of genes. Cancer progression is revealed by many genes, and examples come from:

(i) The presence of interactions involved in core cell-cycle and DNA-damage repair pathways that are significantly rewired in tumors, indicating a significant impact on key genome-stabilizing mechanisms;
(ii) Several flipped genes that are serine/threonine kinases which act as biological switches, reflecting cellular switching mechanisms between stages;
(iii) Different sets of genes flipped during the initial and final stages, indicating a progressive pattern.

The control problem can in general be concentrated on the search for a master regulator of the perturbations affecting potential targets. The principle of structural controllability can thus be used to identify a minimal set of driver nodes that exert control the network states. Since nonlinearity induces dynamics hard to control, the attractor principle and associated landscape analysis can also be usefully considered for differentiating between normal and cancer states and optimize possible control strategies [64,65,66,67]. The co-existence of multiple attractors allows stepping to an attractor network, or equivalently a coarse representation of the system phase space, would then be possible by identifying all possible attractors in the system, and once nodes are assigned to them the gain for control purposes appears from the fact that driving the network to a desired state in a finite time would be more efficiently done [68]. A control kernel would be identified as the minimal set of nodes that once regulated would drive the network from any initial state to any target state [69]. Clearly enough, such kernel inspires the possibility of identifying its components with cellular phenotypes, and possibly with drug targets, and such kernel structure leverage the inherent property of state coherency (distribution over the attractors). 

Since noise is always a possible inducer of transitions between states and network components, a complementary aspect of such developments refers to the role that compensatory perturbations may have to direct the network to a desired state, by taking advantage of its basin of attraction and thus exerting a so-called network reprogramming [70]. This concept refers to seminal work on survival signaling in lymphocyte leukemia [71,72] and on cancer subtypes [73].

Controllability, namely the ability of a dynamical system to step between states, say from an initial to a final state in finite time, can be verified quite straightforwardly in linear time-invariant systems by the so-called Kalman’s rank condition [74]. Given a canonical system:

dz = Az + Bv(11)

with state vector z, and A and B as state and control parameter matrices, respectively, the reference condition is:

rank{B,AB,……, A^P−1^ B} = P (12)

Naturally enough, things become more complicated when the system’s parameters are unknown. Furthermore, a common strategy to establish network controllability turns to the identification of a minimum set of driver nodes. This implies that controlling the set is equivalent to controlling the entire system [75,76,77], and thus the problem in applications is to achieve the highest possible controllability or alternatively the controllability enabled by lowest possible number of key nodes. Thus, the dynamics relevant to controllability refer to selected node densities through which the influence is exerted at global scale [78]. This is coherent with the fact that the real network complexity may be reduced to a context considering node-specific hidden variables that once transformed may reveal latent symmetries. If at each network N is assigned a probability p(N), then statistical ensembles of graphs can be designed by considering families of such stochastic networks [79]. These will be stochastically symmetric under a transformation if each member network has the same property under the same transformation. An entropy optimization problem is then presented when searching for the maximum entropy probability. This problem usually implicates the existence of a topologically constrained network, particularly when the ensemble network plays the role of null model.

## 3. Results

The following use cases refer to recent studies centered on metastatic cancers analyzed through various methodologies delivering differentially expressed gene (DEG) profiles. 

### 3.1. Use Case 1: Entropic Patterns in Metastatic Breast Cancer

**Figure 1:**
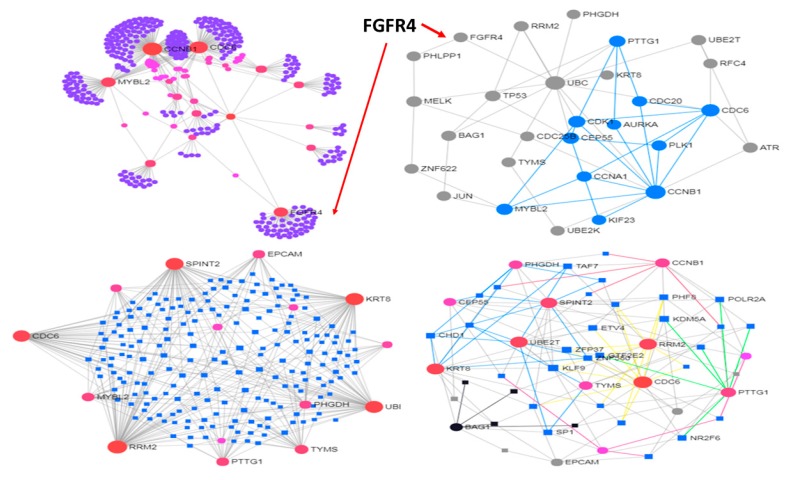
Protein Interaction Networks (PIN) from 16 metastatic over-expressed genes in BC. Top panel: Left, whole network; Right, modular map of reduced sub-network. Bottom panel: left, whole TF-gene network; Right, modular map of reduced sub-network.

**Figure 2:**
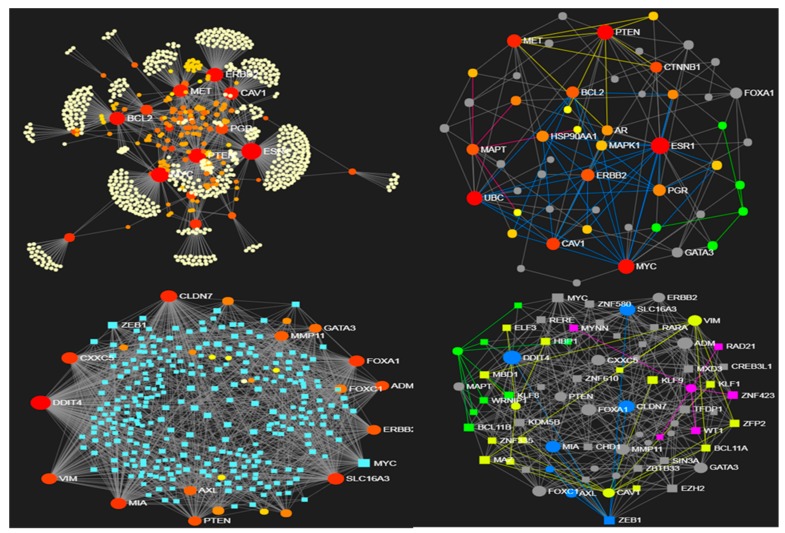
PIN from 31 metastatic under-expressed genes in BC. Top panel: Left, whole network; Right, modular map of reduced sub-network. Bottom panel: left, whole TF-gene network; Right, modular map of reduced sub-network.

From the data obtained with 123 paired primary and metastatic tissues in breast cancer patients [80], the networks displayed in Figure 1 and Figure 2 were built. Overall, 47 genes (16/31 genes significantly over/under-expressed) were found differentially expressed in the metastatic cases. The protein-protein interaction networks (source STRING db, and confidence level fixed at relatively high threshold, namely 0.7) are shown for the over-expressed DEG proteins (375 nodes and 476 edges) and for the under-expressed DEG proteins (772 nodes and 1113 edges). Due to network size, the minimum connected (reduced) network configurations are also computed (28 nodes and 52 edges, and 53 nodes and 141 edges, respectively). 

The modules of the reduced networks were then computed using preferentially the algorithm *WalkTrap* [81] (*Label Propagation* [82] and *InfoMap* [83] were applied too for comparative evaluations). Another constraint for these data is provided by the gene regulation that transcription factors (TF) induce (ENCODE Chip-seq data is the source db). The network of the over-expressed genes includes 217 nodes and 413 edges (reduced to 51 nodes and 136 edges when the minimum connected network is considered), and is dense in communities (each employed algorithm finds substantial modularity, but in the reduced network only *WalkTrap* detects six modules). With the under-expressed genes the TF-gene network includes 313 nodes and 1208 edges, while the reduced network has 73 nodes and 354 edges (here the *WalkTrap* algorithm detects four modules). 

The above networks have represented the breast cancer (BC) data state space subjected to constraints, i.e., the sign of gene differential expression and the retrieved interactions centered on TF regulation over their target genes. Modularity represents also a topological constraint applied to these networks, which explains the presence in both original and reduced networks. Overall, the metastatic profile here analyzed reveals structure details that are organized in modules, hence they imply specific functional activity. Looking at both over-expressed and under-expressed genes in the metastatic BC profile, a richer modular map appears in the under-expressed fraction of the gene profile, possibly induced by the bigger sub-profile size rather than by inherent dynamics. 

The reference study on BC subtyping reported that large absolute expression changes at the RNA level between primary and metastatic disease were not identified. This lack of differentiation weakens in principle the possibility of defining a constrained state space associated to gene profiles. A pivotal role (top over-expressed gene) was assigned to FGFR4 (fibroblast growth factor receptor 4), a well-known player in ovarian and BC tumorigenesis whose over-expression, especially in BC, characterizes the HER2-E intrinsic subtype. In Figure 1 it shows to be a modular driver at whole network scale (top-left plot), less so at reduced network scale (right-top plot, not part of the circled blue module). Because FGFR4 exerts subtype-specific influence, and this is related to the state of the cells, a lower entropy would be expected due to a decreased number of degrees of freedom in the system. This local entropy decrease could represent a general signature of metastasis, thus mitigating the primary-secondary tumor difference.

In general, constraints in a network are associated with decreased entropy. Network topology establishes several types of constraints. When network re-modulation takes place due to perturbing factors, the effects are expected over DEG profiles, activated pathways and wired modules. Despite it may seem complicated to interpret the entropy changes in a cancer context, higher entropies tend to underlie increased network plasticity. It was reported that over-expressed gene profiles showed reduction of entropy compared to under-expressed gene profiles, and this may indicate superior robustness according to the fluctuation theorem, for instance due to adaptation to the selective pressure of the tumor microenvironment. Conversely, lower entropies typically induced by constraints limit cells state dynamics and reduce the system’s degrees of freedom.

### 3.2. Use Case 2—Controllability in Metastatic Stomach Cancer

**Figure 3:**
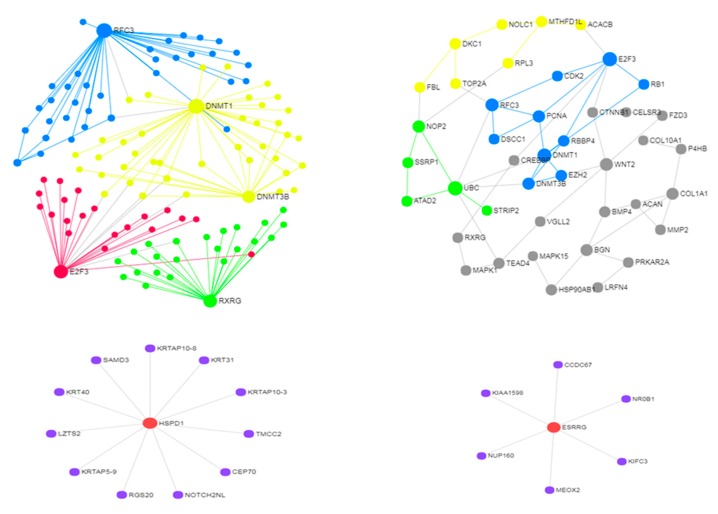
PIN from 21 metastatic and 11 non-metastatic stomach adenocarcinoma genes. Top panel: whole (left) and reduced networks induced by mixed gene group; Bottom panel: over-expressed (left) and under-expressed hubs.

This study was on 21 metastatic samples and 11 non-metastatic samples of stomach adenocarcinoma, and 2979 DEGs were obtained in the first group and 491 in the second group [84]. In the first DEG profile, 1251 were over-expressed genes and 1728 were under-expressed genes, with 2593 genes tissue-specific. Other 386 DEGs appeared in both groups showing similar expression patterns. Here, the top-50 DEGs in this minor sub-profile were used to build a network (Figure 3, top panel). We can see that, despite the lack of differentiation, these gene sub-profile reveals modular structure at both large and small scale, i.e., after suitable network reduction (42 nodes and 58 edges). 

When we consider specific metastatic sub-profiles (top-ten overexpressed and under-expressed DEGs), the induced small networks are not modular and centered on only two hubs, *HSPD1* (over-expressed) and ESRRG (under-expressed). This is further confirmation that the topological constraint of modularity is not due necessarily to the metastatic characteristics. A substantial degree of entropy in the system remains likely non-associated to specific phenotypes. For instance, the subtypes in this cancer that are well-known and usually investigated are a few, say four. Nevertheless, *HSPD1* and *ESRRG* are considered promising metastatic markers for this cancer, explaining one how the cancerous cells escape from apoptosis and being the other a component of a five-gene gastric cancer signature, therefore an immediate target at both clinical and network (controllability) levels. 

### 3.3. Use Case 3—Symmetry and Synchronization in Melanoma State Transitions

In a recent publication on melanoma [85], the metastatic process was analyzed through the transcriptional reprogramming that occurs during the transition from proliferative to invasive states. Many genomic regulatory regions were revealed for the melanoma states from joint transcriptome and methylome profiling, and master regulators were identified in *SOX10/MITF* (*SOX-10* is a marker for melanocytic differentiation, and its dysfunction impairs MITF, the Microphthalmia transcription factor expression, as well as melanocytic development and survival), and AP-1/TEAD (together, they critically regulate transcriptional and functional mechanisms in cancer cells, particularly invasion and resistance [86]). 

**Figure 4:**
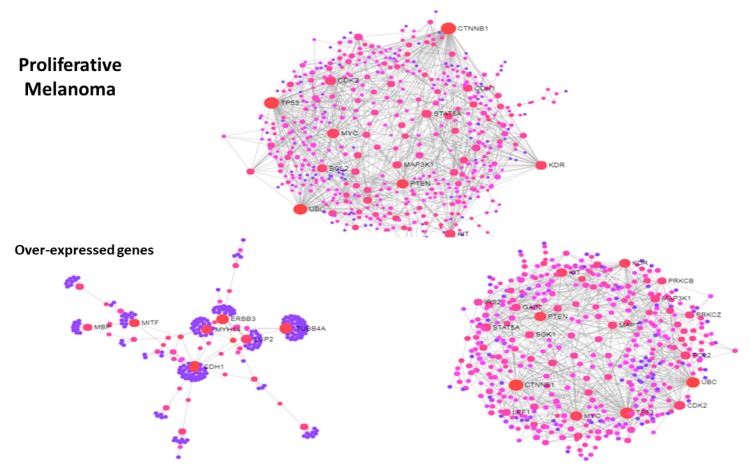
PIN from proliferative DEG signature in melanoma (left panel). Top: whole network. Bottom: reduced networks, with the top 150 over-expressed values (left) and the rest (right).

**Figure 5:**
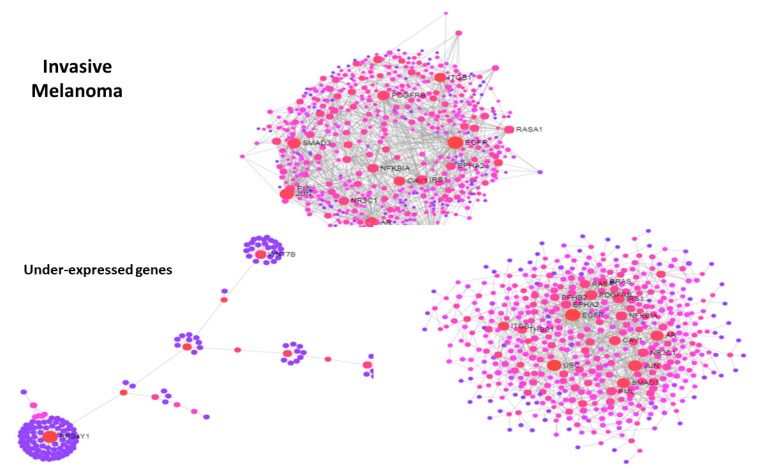
PIN from invasive DEG signature in melanoma (right panel). Top: whole network. Bottom: reduced networks, with top150 down-expressed values (left) and the rest (right).

Figure 4 and Figure 5 show protein-protein interaction networks obtained from two melanoma signatures, proliferative genes (over-expressed) and invasive genes (under-expressed), respectively. Reduced networks were obtained from the top-150 over-expressed (proliferative DEGs) and the top-150 under-expressed (invasive DEGs) values. The two global signature networks are dense and present key hubs, namely PDGFRB, ITGB1, EGFR, NFKBIA, JUN in invasive, and CDK2, STAT5A, TP53, MYC, BCL2, PTEN, MAP3K1, KDR, KIT in proliferative, and many communities were found. To understand the origin of modularity, a split was operated between the top-150 DEGs in both signatures and the rest of DEG values, and their networks were plotted. In both signatures, particularly in the proliferative one, the contribution to modularity of the top DEGs is evident compared to the other DEG values, indicating that the strongest signals drive the interaction dynamics modularly especially with proliferative genes. This transition between proliferative and invasive cells is a sign of disease progression that connects metastasis to the presence of community structure.

**Figure 6:**
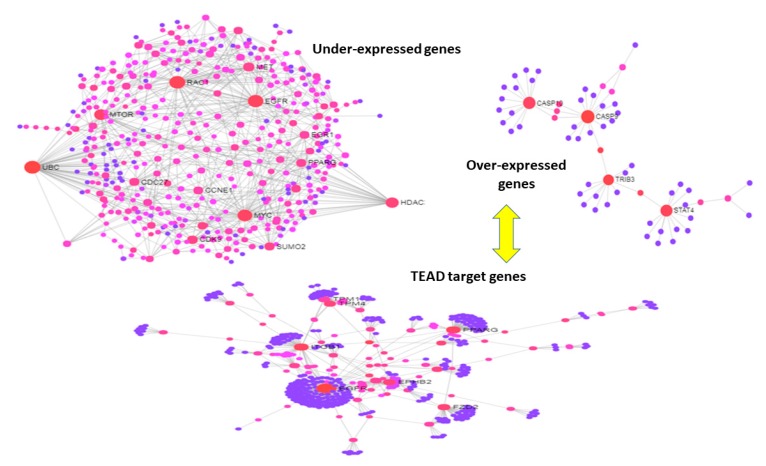
PIN from TEAD knockdown experiments. Top-left under-expressed DEG values; Top-right: over-expressed DEG values. Bottom: TEAD target DEG values.

Further evidence is obtained with other sets of DEG values. For instance, following TEAD knockdown experiments, Figure 6 shows at one hand the under-expressed DEG values as dense and bulky (left plot) while the over-expressed DEGs as sparsely modular (right plot), while the set of the TEAD target DEG values with expression log2fc > |1| is highly modular (bottom plot). This seems to indicate that a strong influence on modularity is induced by TEAD through only its target genes, i.e., just a subset of the DEG sets that were considered. Therefore, acting over the target genes by TEAD knockdown changes the network configuration resolving part of its inner modularity or in other terms relaxes a system’s (topological) constraint. More importantly, the translational medicine impact of modularity acting as a metastatic signature is reinforced by observing the knockdown-driven (intervention) effects that possibly coordinate symmetry and synchronization dynamics at a diffuse network regulation scale. 

## 4. Discussion

Due to the importance of state transitions in cancer networks, many dynamic properties that are currently analyzed through entropy, symmetry and controllability can become interpretable from a biological and, hopefully in the future, clinical standpoint. Among the most important network states exerting influences, the attractor states are stable and can be considered differentiated cell types, thus also phenotypes such a subtypes or tumor cell characteristics. We know that cell state transitions have associated patterns of gene expression profiles, and from such patterns we can investigate network configurations and assess inference quality (optimal use of algorithms and results interpretability).

A system likely spends most of the time within the basins of attraction, and relatively little time moving between them. This depends on the fact that stationarity tends to recur and non-stationarity transiently breaks it. Underlying both states there are differentiation processes whose stochastic dynamics can be tracked by monitoring the inter-state trajectories covering different regions of the state-space. The activation of the states is the element inducing the observed changes, therefore the measurable trajectories. The signaling activity patterns measured by gene profiles and by the related pathways may be cast within network representations to allow more dimensions and also more constraints to be investigated through the topological properties.

The control of a large dynamical complex network may be a very hard task to accomplish in biomedical applications [87,88,89,90]. It might be intuitively efficient to control the state of subsets of nodes instead of single nodes, but identifying functional sets as markers or targets is a highly context-specific problem that requires complex algorithms rather than simplified solution paths. The identification of symmetries in a complex system can be very important in order to decipher its governing organizational principles and rules. The key problem is to understand the role of symmetries in reconstructing or controlling network dynamics. 

Naturally enough, it is important to compute an optimal network decomposition into observable/controllable and unobservable/uncontrollable sub-networks likewise into symmetry-driven versus non-symmetry-driven sub-networks to study how they synchronize or desynchronize. The problems remain harder in nonlinear networks with symmetries, due to the fact that observability and controllability present more complicate dependence relationships.

## Materials and Methods

The datasets used for the application section and the described examples have been retrieved from published literature sources reported in each use case. Network software sources include the iGraph R package (among other ones publicly available, such as WGCNA, network etc.) and visualization tools include cytoscape tools (networkinference) and r-graph-gallery (network). PIN were created from standard annotation sources (string-db).
